# Leveraging Remote Research Associates During a Pandemic

**DOI:** 10.5811/westjem.2020.6.48043

**Published:** 2020-07-21

**Authors:** Alexandrea O. Cronin, Morgan A. Carlile, Christian J. Dameff, Christopher J. Coyne, Edward M. Castillo

**Affiliations:** * University of California San Diego, Department of Emergency Medicine, San Diego, California; † University of California, San Diego, Department of Biomedical Informatics, San Diego, California; ‡ University of California, San Diego, Department of Computer Science and Engineering, San Diego, California

## Abstract

**Introduction:**

The coronavirus disease 2019 (COVID-19) pandemic has seriously impacted clinical research operations in academic medical centers due to social distancing measures and stay-at-home orders. The purpose of this paper is to describe the implementation of a program to continue clinical research based out of an emergency department (ED) using remote research associates (RA).

**Methods:**

Remote RAs were trained and granted remote access to the electronic health record (EHR) by the health system’s core information technology team. Upon gaining access, remote RAs used a dual-authentication process to gain access to a host-based, firewall-protected virtual network where the EHR could be accessed to continue screening and enrollment for ongoing studies. Study training for screening and enrollment was also provided to ensure study continuity.

**Results:**

With constant support and guidance available to establish this EHR access pathway, the remote RAs were able to gain access relatively independently and without major technical troubleshooting. Each remote RA was granted access and trained on studies within one week and self-reported a high degree of program satisfaction, EHR access ease, and study protocol comfort through informal evaluation surveys.

**Conclusions:**

In response to the COVID-19 pandemic, we virtualized a clinical research program to continue important ED-based studies.

## INTRODUCTION

Academic medical centers across the world are actively involved in clinical research and their success and quality relies on the collaborative contributions of all levels of its research staff. Emergency departments (ED) often use volunteer research associates (RA) to support clinical research activities such as study screening and enrollment while providing students with clinical research experience.^1^,^2^

In response to the declared coronavirus disease 2019 (COVID-19) pandemic^3^ and in adherence with state and local restrictions,^4^,^5^ operations within academic medical centers have been significantly impacted to ensure patient, staff, student, and volunteer safety. Clinical research tasks are necessary even during the pandemic to evaluate new protocols and treatment options, enroll new patients in ongoing studies, conduct follow-up on existing patients, and still have access to high quality and peer-reviewed data in well-designed trials. This pandemic has modified how we think of patient care and conducting research from afar beyond traditional remote chart review. Advances in technology have allowed for the remote care of some patient conditions and supportive activities such as data reporting. We hoped to expand prospective research activities using remote access, which is not as prominent in the literature.

Advances in electronic health record (EHR) capabilities have transformed how we capture, store, and summarize patient data.^6^ Equally important technology advances, such as security protocols and virtual networks, have allowed clinicians and researchers to access information securely and remotely. Cybersecurity specifically allows for authorized access to an established system while preventing unauthorized access. Given the abundance and importance of clinical research to improve the quality of patient care and continue educational objectives of health systems, we have successfully implemented and positioned remote RAs to continue ED-based research objectives during the COVID-19 pandemic. The purpose of this paper is to describe the implementation of a program to continue clinical research based out of an ED using remote RAs.

## METHODS

This paper describes a remote RA program developed at an academic medical health system with two hospital EDs using a shared EHR (Epic) with a combined census of 85,000 patients annually. One hospital is an urban, academic teaching hospital (a Level 1 trauma center with the region’s only burn center) and the other is a suburban community hospital with a geriatric emergency care unit within the ED. The health system has extensive multidisciplinary research programs that rely on standard clinical research methodologies to conduct innovative research studies and clinical trials.

The University of California San Diego Department of Emergency Medicine RA program was established in 2002 to provide RAs with the opportunity to gain clinical research experience in the ED while receiving academic and professional mentorship. There are usually 40–50 RAs in the program at a time who cover about 16 hours in both EDs per day. Prior to COVID-19, RAs provided active engagement with ED patients to collect data for numerous ongoing studies and support other research opportunities. Many students obtain independent study college credit for their participation in the program.

All RAs are required to complete Human Subjects Protections (HSP) training and EHR training. HSP training is conducted through the Collaborative IRB Training Initiative (CITI) and set forth by the institution’s expectations. RAs must also complete Epic ASAP EHR Training. Next, RAs are scheduled for a one-hour orientation with a faculty or staff member to understand existing ED-based study needs and departmental expectations. RAs then shadow a training officer in the ED and complete a competency checklist that reinforces program expectations in order to be able to serve shifts independently. Time with the training officer varies based on individual RA level of comfort and ED-specific factors such as patient availability.

Providing remote access to a group of existing RAs allowed for two specific studies to continue, while allowing RAs to gain clinical research experience and independent study credit. Student participation in these studies requires skills to complete tasks beyond remote chart review. One of the projects is an observational study that involves the evaluation of a rapidly implemented clinical care pathway to treat COVID-19 patients that began following the implementation of the stay-at-home measures; the other project is an ED-based, randomized controlled trial to refer to and assess two palliative care treatment arms for patients who had been previously diagnosed with a life-limiting illness. In support of these two studies, RAs attended a remote one-hour training session for each study provided by each study’s principal investigator and RA program administrators that involved a live demonstration and discussion of study-specific inclusion and exclusion criteria.

The week following the initial remote setup, a program coordinator conducted 30-minute scheduled remote consultations with individual RAs to review study criteria, troubleshoot remote access issues, and informally assess individual RA competence of study-specific expectations. To informally assess RA competence of study-specific expectations, we asked questions about navigating the EHR and other important study-specific processes. We summarized all program and study training time allotments in Table 1. Roughly seven weeks following the implementation of remote RA access, we informally assessed the perceptions of the advantages and disadvantages of conducting remote research from six RAs. Responses from the informal assessment are summarized in Table 2.

Expanding on existing network infrastructure protected by a host-based firewall, six existing RAs were provided guidance and training on dual authentication processes and access to a virtual private network (VPN) from which they could access the EHR. Access to the VPN is granted using a single sign-on (SSO) process in an active directory that can only be modified by core information technology (IT) team members. These RAs independently established access to a two-factor authentication system using unique credentials to access the VPN.

## RESULTS

Leveraging skills and resources from research, IT, and clinical staff, we successfully provided access to an EHR via existing network infrastructure to enable six remote RAs in one week. Given that this research program would have otherwise been terminated due to COVID-19, RAs self-reported a high degree of program satisfaction, remote EHR access ease, and comfort with study protocol during the 30-minute check-ins with the program coordinator. For the COVID-19 pathway study, enabling remote RAs allowed them to screen for eligible study participants in the EHR and collect data via telephone from ED attending and resident providers. While there was concern regarding interrupting emergency provider workflows, emergency physicians were receptive to study participation and would communicate at a later time with our RAs if they were busy. For the second study, RAs not only screened for eligible ED patients in the EHR during the patient’s stay, but also worked with the clinical care teams via telephone to confirm subject eligibility in adherence with defined study inclusion criteria. Using details from the EHR, the RAs contacted patients during their ED stay using the patient’s cell phone number or by calling the phone in the patient’s assigned room, conducted informed consent processes via telephone, as deemed appropriate by the IRB, and collected baseline study details.

Overall, our remote RA program success is indicated by our ability to continue collecting data and enrolling patients in our two ED-based studies, as well as our informal assessment of the advantages and disadvantages of conducting research remotely, as summarized in Table 2. Further expansion to other observational and prospective studies and increasing research capabilities, including expansion to remote RAs not tied to college credit, with additional students is moving forward as well. Similar to our site’s ability to enroll patients for this study telephonically through remote RAs, other participating sites in the palliative care study have also been able to continue to enroll patients.

## DISCUSSION

This remote RA approach allowed us to both initiate and continue important research studies to address the effects of a pandemic, but could also be considered as a means to improve efficiency in the future. Similar to how many healthcare settings have responded to the pandemic by reducing or eliminating visitors to reduce the spread of the virus,^2^ the remote RA model reduces the number of people who need to be physically present in the healthcare setting, which is beneficial for patients who may feel overwhelmed by the ED environment and further allows for the optimization and prioritization of their care. This approach opens the realm of clinical research to new opportunities.

Despite the challenges that this worldwide crisis has caused, clinicians and researchers still have an opportunity to respond by rethinking the way they continue prospective clinical research. Involving RAs in research remotely fills an important gap while diversifying and expanding experiences and possibilities for RAs with underlying health conditions to gain experience beyond chart review alone. This is especially true of the COVID-19 pandemic or of similar situations where access to a clinical setting may be detrimental to an individual’s health. While this approach has positive implications for reducing the need for unnecessary exposure and personal protective equipment, it will reduce the ability of study team members seeking in-person, clinical exposure to gain necessary contextual experience. Weighing the pros and cons of this type of approach is thus important and could be cascaded into alternative research experience models.

This approach also changes the way that researchers and clinicians think about subject recruitment and enrollment. While this is currently serving as a temporary alternative to existing workflows, remote EHR access and enrollment by telephone allow research teams with enrollment or other expertise to engage patients from afar, conduct research with special populations, or conduct remote follow-up activities. Similarly, this approach transforms the need for brick and mortar structures for research teams and allows the option to work from a convenient location or designated space. However, this approach minimizes or eliminates the personal contact of in-person subject recruitment.

Finally, leveraging remote RAs expands research teams to include appropriately trained students, off-site clinicians, and other research team members. Study teams are increasingly comprised of research sites or members from various locations to improve sample size and subject recruitment. Leveraging remote RAs could expand the ability of sites to more broadly share information and data with one another – while engaging with a diverse group of study team members – contributing to the improved quality and efficiency of multisite study recruitment, collaboration, and data-related efforts. Our site is one of many sites currently participating in the ED-based palliative care study and leveraging remote RAs to help with research activities for this study has helped to expand study screening and recruitment activities from afar.

## LIMITATIONS

The RAs involved in our program had previously been trained in person on ED workflows and program expectations. In the event that someone without an existing program wanted to start one in a similar way, additional training considerations would need to be considered. For example, our RAs already understood ED workflows, processes and expectations, so transitioning them to remote access was not as challenging.

## CONCLUSION

As technology improves and broadens academic medical centers’ research methods, alternative approaches may enable research continuity, even in a pandemic. While some studies may not be suitable for remote RAs, some projects may continue, and lessons from COVID-19 may be carried forward beyond the pandemic.

## Figures and Tables

**Table 1:** Training types with time allocations for student research associates and faculty/staff.

Training type	Student time allocation (hours)	Faculty or staff time allocation (hours)
CITI HSP and GCP training*‡	4–6	0
EHR training*‡	1–2	0
Orientation**‡	1	1
Shadowing in the ED**‡	2–4	0
Remote access training and study-specific training		
Study 1*	1	1
Study 2*	1	1
Check-ins*	0.5–1	0.5–1

* Training conducted remotely

** Training conducted in person

‡ RA program onboarding (students had completed previously)

*RA*, research associate; *CITI*, Collaborative Institutional Review Board Training Initiative; *HSP*, Human Subjects Protections; *GCP*, good clinical practice; *EHR*, electronic health record; *ED*, emergency department.

**Table 2:** Response themes of remote research associates’ self-reported advantages and disadvantages of conducting remote research.

Advantages	Disadvantages
Having your own (unshared) space Having constant/direct computer access Schedule flexibility More exposure to more studies* Not being exposed to COVID-19 Being able to continue gaining research experience Not needing to consider transportation arrangements Being able to still work with patients	Sometimes challenging to reach busy clinicians Not being in person with patients to gauge indirect communication cues Discussing sensitive topics with patients via phone Patients not understanding why we aren’t there in person Increased challenge of getting the information you need from a patient or clinician Not getting to be in the ED Reaching patients can be tough

* Note: Some remote RAs previously had limited study participation.

*COVID-19*, coronavirus disease 2019; *ED*, emergency department.
